# Prognostic Value of Circulating MG53 Levels in Acute Myocardial Infarction

**DOI:** 10.3389/fcvm.2020.596107

**Published:** 2020-10-28

**Authors:** Hongyang Xie, Zijun Yan, Shuo Feng, Tianqi Zhu, Zhengbin Zhu, Jingwei Ni, Jun Ni, Run Du, Jinzhou Zhu, Fenghua Ding, Shengjun Liu, Hui Han, Hang Zhang, Jiaxin Zhao, Ruiyan Zhang, Weiwei Quan, Xiaoxiang Yan

**Affiliations:** ^1^Department of Vascular & Cardiology, Ruijin Hospital, Shanghai Jiao Tong University School of Medicine, Shanghai, China; ^2^Institute of Cardiovascular Diseases, Shanghai Jiao Tong University School of Medicine, Shanghai, China

**Keywords:** acute myocardial infarction, risk stratification, biomarker, prognosis, MG53

## Abstract

**Background:** Mitsugumin 53 (MG53), a muscle-specific protein belonging to the TRIM family, has been demonstrated to protect the heart against oxidative injury. Although previous studies indicated that ischemic hearts released MG53 into circulation in mice, its effects in humans remains unknown. We aimed to evaluate the prognostic value of MG53 in patients with ST-segment elevation myocardial infarction (STEMI).

**Methods:** Serum levels of MG53 were measured in 300 patients with STEMI, all patients were followed for 3 years. The primary endpoint was major adverse cardiovascular events (MACE), defined as a composite of cardiovascular (CV) death, heart failure causing-rehospitalization, recurrent myocardial infarction (MI), and stroke.

**Results:** Patients with a higher concentration of serum MG53 tended to be older, with a history of diabetes. MG53 levels were also highly associated with indicators reflecting heart function, such as left ventricular ejection fraction (LVEF), N terminal pro B type natriuretic peptide (NT-pro-BNP), and cardiac troponin I (cTnI) at baseline. Kaplan-Meier survival curves demonstrated that patients with MG53 levels above the cutoff value (132.17 pg/ml) were more likely to have MACEs. Moreover, it was found to be a significant predictor of CV death (HR: 6.12; 95% CI: 2.10–17.86; *p* = 0.001). Furthermore, the C-statistic and Integrated Discrimination Improvement (IDI) values for MACEs were improved with MG53 as an independent risk factor or when combined with cTnI.

**Conclusions:** MG53 is a valuable prognostic marker of MACE in patients with AMI, independent of established conventional risk factors, highlighting the significance of MG53 in risk stratification post-MI.

## Introduction

Myocardial infarction is the most common cause of high mortality in the modern world (1). Despite all the improved treatments, the prognosis of patients with acute myocardial infarction (AMI) is still poorer than that of most other diseases, especially in the aging population (2). Complications of AMI, such as heart failure and cardiovascular (CV) events, could further affect the quality of life for patients (3, 4). Therefore, identification of patients with poor prognosis may help in the optimization of therapy and outcomes. However, the ability to identify risk-prone patients remains limited, and further exploration of novel biomarkers may help identify risk-prone patients who might benefit from intensified treatment. Studies of molecules that are activated during AMI could also potentially delineate novel therapeutic targets and be of importance for the development of personalized medicine.

Mitsugumin 53 (MG53) was first cloned in a proteomic library to identify proteins associated with myogenesis and muscle integrity (5, 6). It is a relatively novel tripartite motif protein belonging to the TRIM family. These proteins play a crucial role in a wide list of biological processes, such as cell-cycle regulation, oncogenesis, and innate immunity (7–9). Similar to its family members, MG53 has a typical tripartite motif that consists of a ring, B-box, and coiled-coil regions and a C-terminal PRY-SPRY domain. The biological function of MG53 was first reported in 2009 (10). It is essential for the sarcolemmal membrane repair process and participates in the maintenance of skeletal plasma membrane integrity. Moreover, MG53 is related to the insulin signal pathway (11).

A series of studies have shown that MG53 plays a role in myocardial protection against ischemia-reperfusion injury as a potential cardioprotective protein. The function of MG53 in the myocardium was first discovered by Cao et al. (12), who reported that decreasing MG53 could aggravate cardiac damage after IR injury, whereas increasing MG53 protects cardiomyocytes against oxidative injury. Furthermore, injection of recombinant MG53 can moderate cardiac ischemia injury, indicating that secreted MG53 also plays an important role in cardioprotective effect (13).

It has been reported that the serum concentration of MG53 is elevated in humans with type 2 diabetes mellitus and obesity (14). However, almost all studies on the function of MG53 in the heart were achieved in animal models. The clinical value of MG53 in AMI or any other cardiac-ischemia condition is not well-understood in humans. Moreover, it is not well-known whether MG53 could predict the prognosis of patients after myocardial infarction. Therefore, the current study aimed to determine the relationship between circulating MG53 and outcomes of AMI patients.

## Methods

### Subjects

The cohort study enrolled consecutive patients who were referred for coronary artery angiography from July 2016 to June 2017 at the Shanghai Jiao Tong University School of Medicine affiliated with Ruijin Hospital. Inclusion criteria were patients admitted for primary ST-segment elevation myocardial infarction (STEMI). The diagnostic criteria for STEMI were in accordance with the publications by the American Heart Association (15): (1) New ST elevation at the J point in two contiguous leads of >0.1 mV in all leads other than leads V2–V3. (2) For leads V2–V3 the following cut points apply: ≥0.2 mV in men, ≥40 years, ≥0.25 mV in men <40 years, or ≥0.15 mV in women. (3) New or presumed new LBBB or isolated posterior MI. Moreover, all enrolled patients were over 18 years old. Exclusion criteria included patients with severe physical disability or other serious diseases such as malignant tumors and autoimmune diseases. The research was approved by the Institutional Review Board of Ruijin Hospital, Shanghai Jiao Tong University School of Medicine. Each subject provided written, informed consent before enrolment.

### Outcomes

All patients were scheduled to be followed up for 3 years until death or to the last visit. The follow-up data were available for 97.3% of the included patients. The primary endpoint was MACE, consisting of cardiovascular (CV) death, heart failure causing-rehospitalization, recurrent MI, and stroke. Survival date and information about the cause were documented every 3 months, which was mainly identified by regular follow-up clinic or through contact with patients' families.

### MG53 Measurement

Blood samples were collected after diagnosis and before PCI. Whole blood samples were then centrifuged at 2,000 rpm for 15–30 min to acquire serum. After centrifugation, serum samples were stored at −80°C until use. The concentration of MG53 was measured using Human-MG53 ELISA kits (Cat# CSB-EL024511HU). All samples were measured in duplicates, blinded to patient information.

### Echocardiography

Echocardiography was performed by the same experienced investigator during hospital admission. The LVEF was calculated using the biplane Simpson method in two-dimensional apical four-chamber views.

### Statistical Analysis

Continuous variables are summarized as the mean ± SD or median ± interquartile range where appropriate, and were compared using independent student's *t*-test, one-way ANOVA or Kruskal-Wallis *H-*test where appropriate. Categorical variables are expressed as percentages and frequencies of the cohort and were compared using the chi-squared test. To visualize the relationship between MG53 and survival data, Kaplan-Meier plots were constructed. Univariate and multivariate Cox proportional hazards regression models were used to assess the association of MG53 with composited outcomes. The additional predictive new predictor over a reference model was assessed using Harrell's C-statistics calculated from a Cox proportional hazards regression model (16). The *P*-value of the C-statistics and IDI compared with the reference model was performed by a likelihood ratio test used for the Cox models (17).

To best predict MACE in all patients, we used the cutoff value of MG53. To estimate the cutoff, R software (version 4.0.2; R Foundation for Statistical Computing, Vienna, Austria) was used. All statistical analyses were performed using SPSS software (version 23.0; SPSS, Inc., Chicago, IL, USA). Statistical significance was considered as 2-tailed, *P* < 0.05. All authors had full access to all the data in the study and took responsibility for the integrity of data and accuracy of data analysis.

## Results

### Baseline Characteristics

A total of 300 STEMI patients were enrolled in this study; the mean age of patients was 65.39 ± 12.57 years, and 79.3% were male. All demographic data of patients were divided into three groups depending on the tertiles of MG53 level for further analyses. Table 1 shows tertiles of MG53 levels with respect to baseline characteristics, comprising clinical data found during hospitalization and at admission. When stratified by the onset time of chest pain, MG53 levels were highest at 12–24 h (Table 2).

**Table 1:** Baseline characteristics of patients with STEMI by tertiles of MG53.

	**MG53≤74.08 pg/ml**	**74.08 < MG53≤106.78 pg/ml**	**MG53>106.78 pg/ml**	***P-*value**
	**(*n* = 99)**	**(*n* = 101)**	**(*n* = 100)**	
**Demographic and risk factors**				
Age (years)	59.08 ± 12.84	66.46 ± 10.72	70.57 ± 11.38	<0.001
Sex (male, *n*, %)	82 (82.8)	74 (73.3)	82 (82.0)	0.179
BMI (kg/m^2^)	24.79 ± 2.69	24.068 ± 2.75	23.42 ± 3.33	0.004
Heart rate (beats/minute)	80.38 ± 12.72	80.25 ± 14.02	88.84 ± 19.55	0.001
Systolic blood pressure (mmHg)	128.42 ± 23.03	120.85 ± 16.76	122.15 ± 21.56	0.036
Diastolic blood pressure	78.43 ± 13.85	73.44 ± 12.50	72.86 ± 13.80	0.018
Alcohol use (*n*, %)	26 (26.3)	22 (21.8)	27 (27.0)	0.652
Smoking (*n*, %)	50 (50.5)	49 (48.5)	37 (37.0)	0.117
**Medical history**				
Hypertension (*n*, %)	51 (51.5)	58 (67.4)	64 (64)	0.204
Diabetes mellitus (*n*, %)	20 (20.2)	24 (23.8)	38 (38.0)	0.012
Dyslipidemia (*n*, %)	26 (26.3)	36 (35.6)	26 (26.0)	0.231
CKD (*n*, %)	6 (6.1)	2 (2.0)	11 (11.0)	0.032
Stroke (*n*, %)	9 (9.1)	5 (5.0)	6 (6.0)	0.476
Prior PCI (*n*, %)	16 (16.2)	5 (5.1)	15 (15.0)	0.031
Prior CABG (*n*, %)	4 (4.0)	0 (0)	3 (3.0)	0.149
**Clinical presentation**				
hsCRP	2.00 ± 3.00	3.08 ± 6.34	9.55 ± 4.97	<0.001
NT-pro-BNP	251.60 ± 585.50	635.00 ± 1588.40	2026.00 ± 4571.50	<0.001
WBC (×10^9^/L)	9.20 ± 3.10	9.00 ± 4.00	11.05 ± 8.25	0.496
Hemoglobin (g/L)	142.00 ± 21.00	135.00 ± 23.00	134.00 ± 30.50	<0.001
Platelet (×10^9^/L)	200.00 ± 63.00	195.00 ± 68.00	180.50 ± 66.30	0.151
Fasting glucose (mmol/L)	5.73 ± 2.12	5.66 ± 1.74	6.82 ± 2.82	<0.001
HbA1c (%)	5.90 ± 0.95	5.90 ± 0.90	6.25 ± 1.42	0.01
Total cholesterol (mmol/L)	4.42 ± 1.40	4.77 ± 1.55	4.50 ± 2.03	0.187
Triglyceride (mmol/L)	1.57 ± 0.88	1.24 ± 0.89	1.27 ± 0.70	0.206
HDL-C (mmol/L)	1.08 ± 0.35	1.05 ± 0.37	1.04 ± 0.31	0.820
LDL-C (mmol/L)	2.74 ± 1.35	3.09 ± 1.15	2.87 ± 1.65	0.087
Creatinine (μmol/L)	75.00 ± 20.00	85.00 ± 23.00	96.50 ± 70.00	<0.001
eGFR (mL/minute/1.73m^2^)	92.25 ± 18.47	78.80 ± 27.70	67.50 ± 52.25	<0.001
Cystatin C (mg/L)	0.97 ± 0.22	1.06 ± 0.45	1.11 ± 0.71	<0.001
CK-MB max (mg/L)	82.90 ± 173.90	102.40 ± 238.60	184.50 ± 276.00	0.002
cTnI (ng/L)	9.84 ± 43.78	20.27 ± 56.73	35.69 ± 78.84	<0.001
Syntax score	17.00 ± 16.50	19.00 ± 14.30	22.25 ± 16.00	0.002
LVEF (%)	60.00 ± 7.00	58.00 ± 11.00	54.00 ± 12.00	<0.001
**Culprit vessel**				
LAD (*n*, %)	47 (47.5)	66 (65.3)	50 (50.0)	
LCX (*n*, %)	15 (15.2)	10 (9.9)	7 (7.0)	
RCA (*n*, %)	34 (34.3)	20 (19.8)	39 (39.0)	
Number of diseased vessels	2.19 ± 0.83	2.2 ± 0.81	2.47 ± 0.79	0.14
**Killip classification**				<0.001
I (*n*, %)	94 (94.9)	84 (83.2)	27 (27.3)	
II (*n*, %)	5 (5.1)	12 (11.9)	40 (40.4)	
III/IV (*n*, %)	0 (0)	5 (5.0)	32 (32.3)	
**Medications**				
Statins (*n*, %)	97 (98.0)	96 (100.0)	91 (95.8)	0.124
ACEI/ARB (*n*, %)	84 (84.8)	86 (89.6)	74 (77.9)	0.084
β-blockers (*n*, %)	94 (94.9)	89 (92.7)	86 (90.5)	0.493
Calcium Channel blockers (*n*, %)	11 (11.1)	9 (9.4)	10 (10.5)	0.922
Nitrates (*n*, %)	31 (31.3)	31 (32.3)	44 (45.8)	0.064

*Values are presented as mean ± standard deviation (SD). MG53, Mitsugumin 53; HDL, high-density lipoprotein; hsCRP, high sensitivity C reactive protein; LDL, low-density lipoprotein; LVEF, left ventricular ejection fraction; NT-pro-BNP, N Terminal pro-brain natriuretic peptide; PCI, percutaneous coronary intervention; and WBC, white blood cell. ACEI, angiotensin-converting enzyme inhibitor; ARB, angiotensin receptor blocker; BMI, body mass index; CABG, coronary artery bypass grafting; CCB, calcium channel blocker; CKD, chronic kidney disease*.

**Table 2:** Concentration of MG53 (pg/ml) in patients with chest pain onset within 24 h.

**Time of chest pain onset**	***n***	**Mean**	**Min**	**Max**	**SD**	**Median**
0 h ≤ T ≤ 6 h	96	100.006	21.684	226.782	46.385	97.031
6 h < T ≤ 12 h	72	106.985	27.679	236.000	49.933	99.180
12 h < T ≤ 24 h	78	116.411	33.332	225.257	41.240	102.686

*T, time; h, hours*.

Compared with lower MG53 levels at baseline in Table 1, higher MG53 levels were associated with higher age, poorer renal function, more serious inflammation, and worse general condition. The levels of MG53 were significantly positively correlated with hsCRP, fasting glucose, cystatin C, and creatinine. It was also negatively correlated with hemoglobin and eGFR. No significant difference between male and female patients was observed in this study. Moreover, lifestyle habits such as drinking and smoking were roughly the same in all groups. The STEMI data, including the culprit vessel and the number of diseased vessels, were also comparable among the three groups. Furthermore, there was no significant difference in patients with a history of hypertension, dyslipidemia, and stroke. However, MG53 levels were associated with previous DM.

### Circulating MG53 Levels Were Significantly Correlated With Cardiac Function and Prognosis in Patients With STEMI

In patients with higher MG53 levels, the presence of cardiac dysfunction was much more than that in the lower level group, as shown in LVEF. Higher MG53 levels were also associated with higher NT-pro-BNP, CK-MB, and cTnI levels. Moreover, patients with higher MG53 levels were more likely to have higher Killip classification.

**Figure 1:**
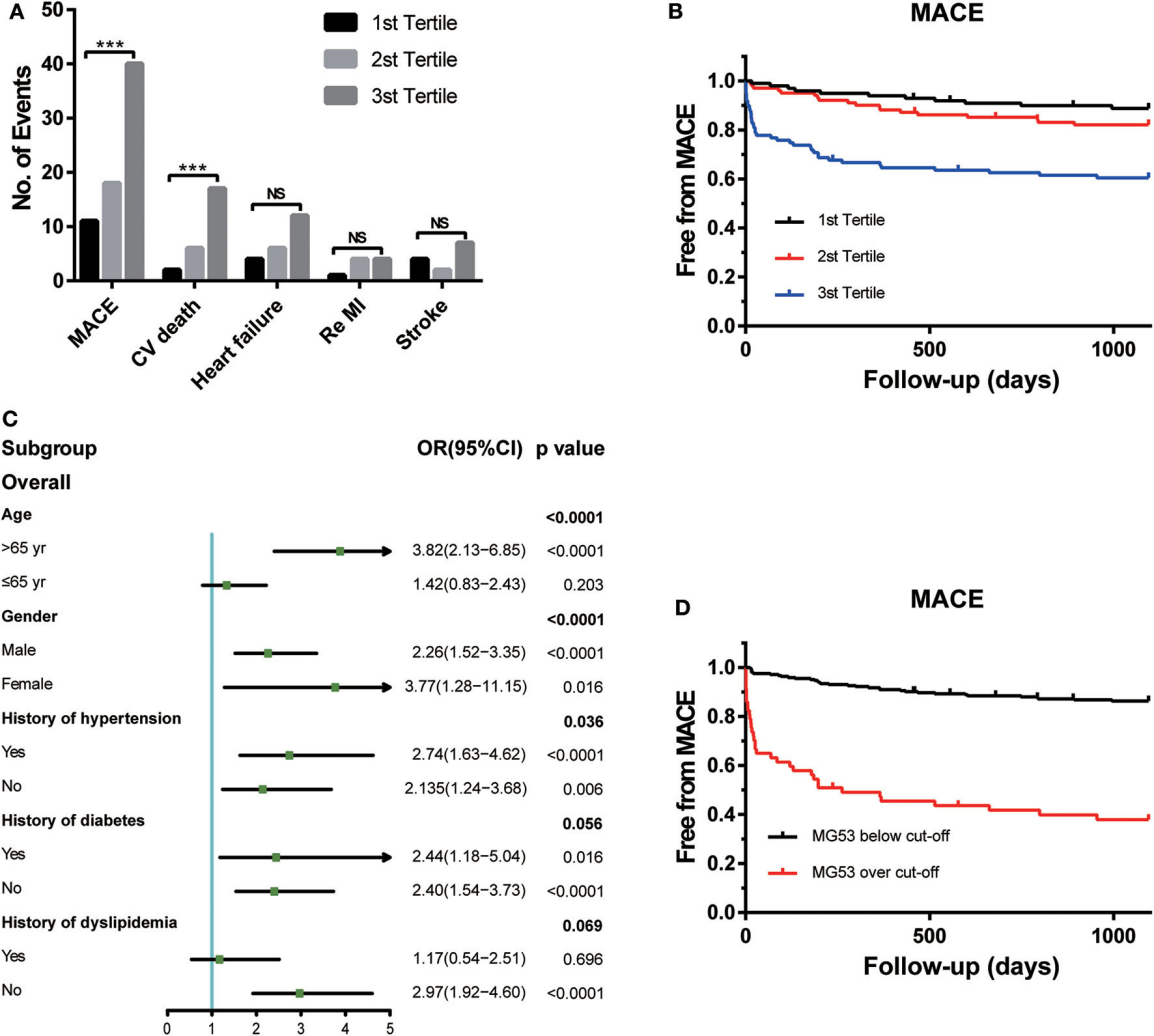
The association between MG53 levels with presence of MACEs. **(A)** Comparisons of the number of events of each outcomes according to tertiles of MG53 levels in the STEMI patients, including MACE, cardiovascular death, HF rehospitalization, re-myocardial infarction, and nonfatal stroke. **(B)** Kaplan-Meier curves for MACE according to the tertiles of MG53. **(C)** Forrest plots to analyze the prognostic value of MG53 for MACE in different subgroups. **(D)** Kaplan-Meier curves for MACE according to the cut-off of MG53. ***p < 0.001, NS, not significant. MG53, Mitsugumin 53.

During the 3 years of follow-up, MACEs occurred in 69 (23%) patients enrolled in the STEMI cohort (Figure 1A). MACEs include CV death, heart-failure rehospitalization, recurrent MI, and non-fatal stroke. Among all these events, the frequency of CV deaths was the highest. The risk of CV death was also positively associated with the tertiles of MG53 levels.

To visualize the relationship between multiple outcomes and different groups of MG53 concentrations, KM survival curves were generated (Figure 1B). Higher MG53 levels were significantly correlated with the presence of MACEs. Moreover, subgroup analysis was performed to assess the predictive ability of MG53 levels in MACEs by using a logistic regression model. As shown in Figure 1C, MG53 levels were a more reasonable prediction in elderly patients regardless of sex. The predictive value for MACEs was also useful in patients with or without concomitant hypertension, diabetes, and dyslipidemia. Furthermore, to best predict the risk of MACEs, the cutoff value of MG53 levels was calculated as 132.17 pg/mL. The KM survival curves indicated a significant difference between the over and below cutoff value groups (Figure 1D).

Cox proportional hazard models demonstrated an independent relationship between MG53 concentration and increased risk for MACEs (Table 3), CV death, and heart failure. Whether converted to logarithmic form or divided into tertiles or cutoff groups, both univariate and multivariable Cox regression analysis can prove the prognostic value of MG53. Moreover, these values persisted after full adjustment for MACEs and CV death. Furthermore, when combined with cTnI, MG53 provided improvement to predict the MACEs in the C-statistic analysis. Similar results were also found in IDI values (Table 4).

**Table 3:** COX hazard models for MG53 with MACEs in patients with STEMI.

	**Unadjusted HR**	***P-*value**	**Adjusted for model 1 HR (CI)**	***P-*value**	**Adjusted for model 2 HR (CI)**	***P-*value**
**MACE**						
Log mg53	4.31 (2.81–6.62)	<0.001	3.83 (2.44–6.01)	<0.001	2.28 (1.44–3.62)	<0.001
mg53 tertiles	2.29 (1.65–3.18)	<0.001	2.01 (1.42–2.84)	<0.001	1.55 (1.08–2.23)	0.018
mg53 1st	Ref		Ref		Ref	
mg53 2st	1.65 (0.78–3.50)	0.19	1.43 (0.67–3.06)	0.36	1.18 (0.53–2.62)	0.69
mg53 3st	4.65 (2.38–9.06)	<0.001	3.81 (1.90–7.62)	<0.001	2.27 (1.11–4.63)	0.025
mg53 cut-off	7.32 (4.55–11.79)	<0.001	6.31 (3.74–10.68)	<0.001	4.32 (2.43–7.67)	<0.001
**CV death**						
Log mg53	8.60 (3.87–19.11)	<0.001	7.85 (3.29–18.73)	<0.001	6.12 (2.10–17.86)	0.001
mg53 tertiles	3.31 (1.77–6.20)	<0.001	2.59 (1.35–4.99)	0.004	1.59 (0.63–3.99)	0.33
mg53 1st	Ref		Ref		Ref	
mg53 2st	3.04 (0.61–15.07)	0.17	2.29 (0.46–11.43)	0.37	1.17 (0.11–11.97)	0.90
mg53 3st	10.48 (2.42–45.39)	0.002	5.98 (1.34–26.75)	0.019	2.34 (0.36–15.15)	0.37
mg53 cut-off	11.41 (5.00–26.07)	<0.001	7.66 (3.21–18.30)	<0.001	6.40 (1.95–20.96)	0.002
**HF**						
Log mg53	3.56 (1.70–7.43)	0.001	3.26 (1.52–6.70)	0.002	1.48 (0.69–3.15)	0.31
mg53 tertiles	2.07 (1.17–3.65)	0.012	1.90 (1.05–3.44)	0.034	1.24 (0.62–2.49)	0.55
mg53 1st	Ref		Ref		Ref	
mg53 2st	1.51 (0.43–5.35)	0.52	1.43 (0.40–5.16)	0.59	1.37 (0.36–5.27)	0.65
mg53 3st	3.87 (1.25–12.02)	0.019	3.29 (1.00–10.84)	0.05	1.55 (0.37–6.42)	0.55
mg53 cut-off	6.77 (2.92–15.67)	<0.001	6.25 (2.47–15.86)	<0.001	2.89 (1.06–7.92)	0.039

*MG53 and biomarkers were transformed into logarithmic form, a categorical variable using the lowest tertile as the reference. MACE indicates a composite endpoint of cardiovascular death, heart failure rehospitalization, recurrent myocardial infarction, or non-fatal stroke*.

*Model 1. adjusted for age and sex; Model 2, adjusted for sex, age, LVEF, diastolic blood pressure, TG, NT-pro-BNP, CK-MB, cTnI, history of hypertension, diabetes, and medications*.

**Table 4:** Accuracy of risk prediction using cTnI, NT-pro-BNP, and MG53.

	**C-statistic**	***p*-value**	**IDI value**	***p*-value**
**MACE**				
cTnI	0.63	–	Ref	–
NT-pro-BNP	0.62	0.72	0.006	0.799
MG53	0.72	0.03	0.153	<0.001
CK-MB	0.60	0.15	−0.017	0.120
cTnI+NT-pro-BNP	0.67	0.11	0.055	0.014
cTnI+MG53	0.73	0.01	0.166	<0.001
cTnI+CK-MB	0.63	0.71	0.002	0.184
cTnI+NT-pro-BNP+MG53+CK-MB	0.72	0.02	0.185	<0.001

*MG53, NT-pro-BNP, CK-MB, and cTnI were transformed into logarithmic form*.

## Discussion

In our present study testing a small population of patients with STEMI, we demonstrated for the first time that serum MG53 levels represent a novel biomarker improving the prognostic value of STEMI patients for composite endpoints. The elevation of serum MG53 before PCI was correlated with cardiac function, which was evaluated by clinical presentation. During long-term follow-up after AMI, the patients with higher levels of MG53 also had a higher risk of MACEs, which enhanced current prediction models. Importantly, the consequences remained statistically significant after fully adjusting for traditional demographic factors and laboratory and imagological examination, including age, sex, LVEF, NT-pro-BNP, CK-MB, and cTnI. Our research indicated that MG53 could augment conventional risk stratification models in patients with STEMI, which may further contribute to the participation of MG53 in human myocardial infarction and its progression.

Cardiac ischemia-reperfusion injury is composed of an immune response, myocardial fibrosis, and ventricular remodeling (18). Because adult cardiomyocytes have a very limited capacity to differentiate or regenerate, ischemia-induced myocardial death leads to local cardiac dysfunction (19), including arrhythmia, heart failure, and even heart rupture. MG53 may participate in acute myocardial infarction and its repair process as an important cardioprotective factor, thus affecting the prognosis.

We found that serum MG53 levels were significantly positively associated with conventional biomarkers such as NT-pro-BNP and cTnI (20, 21), which are released by necrotic myocardium and represent infarct size as well as ventricle function after AMI. The MG53 levels also correlated with the Killip classification identified by the physical signs. These findings demonstrated that MG53 levels could reflect the presence and severity of cardiac injury to some degree. In previous studies, cardiac I/R injury may downregulate the intracellular level of MG53. Lack of MG53 exaggerates the damage, whereas increasing MG53 levels could protect the damaged area against oxidative injury (12). Moreover, S-nitrosylation of MG53 prevented the decrease in MG53 (22). Interestingly, injection of exogenous recombinant MG53 protein can alleviate ischemia damage (23), suggesting that extracellular MG53 also plays a vital role in cardiac protection.

Second, when concentrations of MG53 were divided into three groups by the onset of chest pain, we observed that MG53 levels peaked during 12–24 h after chest pain. This result is in line with previous studies showing that ischemic injury to the heart could lead to the release or secretion of endogenous MG53 into the blood. Liu.et al. found that in mice, a large amount of MG53 could be detected in the cardiac perfusate after I/R injury, and the levels of perfusate MG53 were associated with the levels of creatine kinase (13), supporting the perspective that MG53 levels were correlated with myocardium integrity.

Finally, we also found that circulating MG53 levels were closely related to the LVEF during hospitalization. Moreover, our findings also demonstrated that STEMI patients with higher serum levels of MG53 have lower survival rates. After being fully adjusted for several common risk factors in the Cox regression model, the elevation of MG53 was associated with MACEs and CV death. This means that patients with higher MG53 levels at admission had a higher risk of adverse outcomes. Furthermore, when combined with other biomarkers such as cTnI and NT-pro-BNP, the predictive power for MACEs has improved. This suggests that conventional biomarkers do not fully meet the requirement for risk stratification (24). However, it is unclear whether MG53 levels are associated with poor prognosis and decreased survival rate in STEMI patients. One possibility is that the injured heart releases MG53 to protect itself from IR injury. Based on basic studies, MG53 plays a vital role in ischemia preconditioning and postconditioning protection (12, 25). Both are important protective mechanisms for myocardial injury. MG53 abundance in the circulation could also be upregulated by ischemic preconditioning and anesthetic preconditioning. Hence, maintenance of MG53 concentration is indispensable for cardiac protection. Furthermore, recent studies have indicated that MG53 is expressed in cardiac fibroblasts. MG53 could regulate TGF-β signaling to promote proliferation and migration of fibroblasts (26). These findings may also be involved in the relationship between MG53 and patient prognosis.

## Study Limitations

First, this was a small population cohort study with a single center. The results need further verification in a large population cohort with multiple centers and different genetic backgrounds. Second, the number of adverse outcomes in our cohort was quite small. Thus, we did not have enough information to analyze specific events such as stroke and re-MI.

Moreover, the follow-up time was only 3 years, and further studies with longer-term observation are needed to assess the predictive value of MG53. Third, the MG53 levels at different time points after PCI may be used to improve the predictive ability or accuracy.

Finally, our present research did not prove a direct association between MG53 and cardiac I/R injury. More studies, especially basic research studies, are need to be performed to evaluate the role of MG53 in AMI.

## Conclusions

In the present study, the elevation of serum MG53 levels showed a significant adverse outcome after a 3-year follow-up among patients with STEMI. The measurement of MG53 could be used as a novel biomarker and improving the current means of risk stratification.

## Data Availability Statement

All datasets presented in this study are included in the article/supplementary material.

## Ethics Statement

The studies involving human participants were reviewed and approved by Institutional Review Board of Ruijin Hospital, Shanghai Jiao Tong University School of Medicine. The patients/participants provided their written informed consent to participate in this study.

## Author Contributions

HX and SF collected and analyzed the data. ZY performed the echocardiography. TZ, ZZ, JiN, JuN, RD, JZhu, and FD performed PCI and collected blood samples. HH, HZ, and JZha were in charge of the statistical analysis. XY and HX designed this study and wrote the manuscript. WQ and RZ made critical revisions of the manuscript. All authors contributed to the article and approved the submitted version.

## Conflict of Interest

The authors declare that the research was conducted in the absence of any commercial or financial relationships that could be construed as a potential conflict of interest.
